# Exploring Social Support in an Online Support Community for Tourette Syndrome and Tic Disorders: Analysis of Postings

**DOI:** 10.2196/34403

**Published:** 2022-10-04

**Authors:** Mercédesz Judit Soós, Neil S Coulson, E Bethan Davies

**Affiliations:** 1 Population and Lifespan Sciences School of Medicine University of Nottingham, Queen's Medical Centre Nottingham United Kingdom; 2 National Institute for Health and Care Research MindTech MedTech Co-operative Institute of Mental Health School of Medicine, University of Nottingham Nottingham United Kingdom; 3 Clinical Neurosciences and Mental Health Institute of Mental Health School of Medicine, University of Nottingham Nottingham United Kingdom

**Keywords:** Tourette syndrome, tic disorders, social support, online support communities, online health communities, thematic analysis, online support, peer support, support group, Tourette, online health community

## Abstract

**Background:**

Online support communities have become an accessible way of gaining social, emotional, and informational support from peers and may be particularly useful for individuals with chronic conditions. To date, there have been few studies exploring the online support available for tic disorders, such as Tourette syndrome. An exploratory study looking at users’ experiences with using online support communities for tic disorders suggested that members used such communities to share experiences, information, and strategies for tic management.

**Objective:**

To build on these preliminary findings, this study examined the provision of social support in an online community for Tourette syndrome.

**Methods:**

Data were collected from one publicly available online support community for Tourette syndrome and tics, from its inception to December 2019, by randomly selecting 10% of posts and their corresponding comments from each year for analysis. This resulted in 510 unique posts and 3802 comments posted from 1270 unique usernames. The data were analyzed using inductive thematic analysis.

**Results:**

The findings of this study suggest that users utilized the online community as a multifaceted virtual place where they could share and ask for information about tics, unload and share their feelings arising from living with Tourette syndrome, find people facing similar situations and experiences, and freely share the realities of living with Tourette syndrome.

**Conclusions:**

The results complement the findings from a preliminary study and suggest that online support communities have a potentially valuable role as a mechanism for sharing and gaining information on illness experiences from similar peers experiencing tics and can promote self-management of tics. Limitations and recommendations for future research are discussed.

## Introduction

Tic disorders—such as Tourette syndrome and persistent/chronic tic disorder—are noncurable neurodevelopmental conditions characterized by persistent, involuntary outbursts referred to as tics [1]. Tic disorders usually have their onset in childhood: For many, tics tend to decline or disappear in adulthood, while for others, tics persist [2]. Tics are typically rapid and repetitive and can be vocal (ie, sounds) or motor (ie, spasm-like contractions of muscles). For an individual to meet a diagnosis of persistent/chronic tic disorder, 1 or more vocal or motor tics must be present for more than 1 year since onset; for Tourette syndrome, 2 or more motor tics and at least 1 vocal tic must be present [2]. Tourette syndrome affects approximately 1% of the population worldwide and is more common in men [2,3]. A common public misunderstanding is that coprolalia (tics that involve expressing obscene words) is a key characteristic of Tourette syndrome; however, this affects less than 20% of people with the condition during their lifetime [4] but is independently associated with poorer quality of life, tic severity, and a range of additional neuropsychiatric difficulties [5]. 

Tic disorders are impairing conditions that often negatively impact an individual’s quality of life. Both children and adults with tic disorders frequently report social difficulties, such as being stigmatized or their condition affecting their relationship with family and friends [6]. Negative impact on physical health (eg, pain, injury), decreased self-esteem and mental well-being, functional impairment (eg, delayed progress at school), and decreased overall quality of life have been reported [6-8]. People with tic disorders often experience adverse reactions from other people due to their tics, which include receiving unwanted attention, being excluded, or being bullied [9]. A recent meta-synthesis of 10 studies found that young people often felt ashamed and insecure about their tics, while some adolescents reported defending their rights to be “visible” with Tourette syndrome, and some parents tried to hide or justify their child's tics to strangers, creating tension within the family [10]. In the same review, adults living with Tourette syndrome reported negative experiences across the lifespan, including at school and in workplace environments, and negative experiences with treatment, such as perceiving health professionals as lacking sympathy and knowledge. Furthermore, adults with Tourette syndrome have identified loneliness as a common experience, with readily available support often lacking [11]. Parents of children with Tourette syndrome also frequently report social isolation [12] and have emphasized the significance of using support groups as a “bridge to the outside world” [13].

Social support can be considered as “support accessible to an individual through social ties to other individuals, groups and the larger community” [14]. There are many different functions to social support, including emotional support, informational support, and moral support [15]. It can be a valuable resource when coping with and adjusting to chronic conditions and can promote physical functioning [16] and psychosocial well-being [17,18]. Receiving social support from peers with Tourette syndrome has been reported to help reduce isolation, create feelings of acceptance, and help individuals cope with tics—and can help families too [10-13,19]. Social support may also help counteract the impact of tic disorders upon the psychosocial well-being of individuals and their families [20]. Nevertheless, in-person social support is often unavailable for people with tic disorders due to its rarity [11].

Online support communities (OSCs)—also known by other terms including online support groups, online health communities, and online support forums [21]—have been present for at least 40 years [22], with changes in internet access and increased ownership in internet-enabled technologies over time increasing access to such online spaces. Research into online support groups for health issues emerged in the 1990s, with several reviews published in the early 2000s looking at their usage and effects upon health and social outcomes [23-25]. High-quality trials are lacking in this field; findings from a small number of randomized controlled trials of OSCs have reported mixed results on mental health outcomes [26,27]. However, survey and interview-based research with users of OSCs for many different chronic conditions and health issues (eg, self-harm, insomnia, endometriosis, cancers, polycystic ovary syndrome) have identified the ways in which these virtual communities can be helpful (and unhelpful) to people experiencing a chronic condition or health issue as well as their caregivers [28-34]. Commonly reported benefits of OSCs include having an online space to share and find emotional support for psychological responses to health issues from people who understand it through lived experiences; reducing isolation in facing their health issue by connecting and receiving support from nonjudgmental peers; acquiring new knowledge about their health condition to empower and inform self-management, coping strategies and treatment decisions, and how they interact with health care professionals; and a space where they can create their own narrative and story of their health experiences [28-34]. OSCs may be hosted by an organization (eg, on a health charity’s website), on social networking platforms (eg, Facebook, Reddit), or independently of these [22]. These virtual communities can provide an accessible way of gaining social, emotional, and informational support from peers experiencing the same health issue while removing the geographical and temporal barriers to involvement [29,35]. They may be particularly useful for individuals with rare neurodevelopmental conditions, since they provide opportunities to connect with a larger network of peers than they could find locally. Studies have reported that OSC participation has a positive influence on social engagement in children and young people with neurodevelopmental conditions and has potential to facilitate social networking and support [36].

Several studies have examined the types of social support being exchanged within such online communities serving a range of health conditions [19,37-45]. For instance, Coulson and colleagues [39] studied the provision of social support in messages posted to a Huntington disease online community and reported that group members frequently offered informational (advice, referral, teaching, situation appraisal), emotional (relationship, confidentiality, affection, sympathy, validation/empathy, encouragement, prayer, relief of blame), network (access, presence, companionship, express willingness), and esteem (compliment, anchorage) support, while comparatively few offered tangible assistance (perform direct task, active participation). Attard and Coulson [38] looked at the experiences of members of Parkinson disease OSCs and found that participation allowed members to share experiences and knowledge and to develop friendships, as well as helping them cope with the challenges of living with the condition.

Nevertheless, participation in an OSC is not always a positive experience. Compared with identifying the positives, findings are less consistent for disadvantages of online support. Lack of replies may lead members to feel rejected, members may be rude or judgmental to others due to the greater anonymity, misunderstandings are common due to the format of communication, and information overload may be experienced [38,46]. Furthermore, there is a potential for inaccurate or harmful information to be shared, members who share their complications from treatment can cause others to feel anxious, and success stories may inspire jealousy or hostile interactions within the community [46].

To date, there has been little attention devoted to understanding the role of OSCs for tic disorders. One recent exploratory online survey suggested that online support could “bridge the gap” in accessing support across the course of tics and was a platform through which members could share experiences, information, and strategies for tic management [47]. It can be difficult for patients with tic disorders to access specialist tic services for many environmental and systemic reasons, including issues with health care funding, service delays, referral issues, and a lack of trained specialists [48,49]. This means many people with tics and their families are managing this chronic condition independently—with online support groups helping to “fill this gap” in accessing specialist help. Accessing support online from similar peers may impact a person’s illness experience [38]; for people with tic disorders and their families, members reported that participation influenced their decisions about health care, resulted in improvements in psychological well-being, increased confidence, and resulted in greater acceptance of their tics. [47]. Exploring the types of social support provision and communication online may help in understanding the social support needs specific to people with tic disorders and their families, but, as yet, there has been no attempt to understand how social support is enacted within OSCs for tic disorders.

Several studies have taken deductive approaches to analyze and classify posts in OSCs, for example by applying a social support typology such as the Social Support Behavior Code by Cutrona and Suhr [50]. Taking an inductive approach to analyzing data from naturalistic online communication means researchers can approach data without assumptions about what types of social support they expect to be found, with findings being more reflective of the data set [21]. Furthermore, it is uncertain whether findings from inductive studies map to or are dissimilar to findings from studies using deductive approaches. Therefore, in order to address this, our study sought to inductively analyze the content of messages posted in order to identify and describe the provision of social support within an online community for Tourette syndrome and tics.

## Methods

### Data Collection

Data were collected from one publicly available asynchronous online community devoted to Tourette syndrome and tics. Within this online community, “threads” are started by an individual creating a “post.” Other users and the original poster can reply to a post (“comments”), which in turn can be replied to, giving each thread a complex structure. Posts can be text-based or linked to websites and other media (eg, images, videos). The online community has moderators tasked with moderating the community by removing or locking posts or comments if they break the community’s specified rules.

All messages posted since the community’s inception up to December 31, 2019, were eligible to be included in the data set. During this time, a total of 5382 threads were initiated within the OSC. Each thread title was inspected, and spam messages (eg, adverts) were removed, leaving a total of 5105 threads. Each thread was assigned a number, and using a random number generator [51], 10% of these threads from each year were randomly sampled for analysis. This random sampling approach was taken to ensure that threads were not subjectively chosen by the authors and to reduce potential bias from sampling within a specific timeframe [21,38]. From the 510 threads downloaded for analysis, there was a total of 4312 individual messages (ie, 510 initial messages with 3802 replies) posted from 1270 unique usernames. The content of the 510 threads was downloaded into Microsoft Word.

### Ethical Considerations

The study was granted ethics permission from the Division of Rehabilitation Aging and Wellbeing ethics committee at the University of Nottingham (MEDS4008-20-17). The ownership and use of online community content for research purposes are subject to much debate [21]; for this study, previous studies utilizing similar methodologies were used to guide ethical considerations [19,38,44]. The study adhered to ethical guidelines for internet-mediated research developed by the British Psychological Society [52], including anonymizing the name of the online community. No consent was obtained from users to analyze data, as data were taken from one publicly available online community that did not require registration to read or access posts [38]. Usernames were only used to identify the number of unique users included in analyzed posts and were not used in data analysis. Quotes used to illustrate findings were paraphrased to prevent traceability in online searches [21]. Any potentially identifiable information (eg, names of people, places, health care services) was removed from the data.

### Data Analysis

Due to the exploratory nature of the study and the lack of prior research, the data were analyzed using data-driven (inductive) thematic analysis [53]. The first step was getting acquainted with the data: The lead author (MJS) read through the whole data set multiple times. Second, initial short codes reflecting the data were created across the data set using the computer software ATLAS.ti. The third step was generating potential themes by making a list of the initial codes and moving the items around in the list until there were clusters of similar codes. This step was completed combining ATLAS.ti and Post-it notes arranged into sorting piles. These initial themes and subthemes were put in a thematic map and reviewed and refined multiple times. The third author (EBD) reviewed coded data and supported the creation of themes and subthemes. The fourth step was reviewing the themes and ensuring that the themes captured the researcher’s impression of the data and were independent from one another. The fifth step was defining and naming the themes and subthemes after systematically reviewing them. As in the study by Meade et al [42], the thematic map was discussed and refined with the third author before a final version was agreed upon.

## Results

Our thematic analysis generated 4 main themes: (1) “A place to share and ask for information about tics”; (2) “A place to unload my feelings”; (3) “A place where I can find people like me”; and (4) “A place where I can freely share the realities of living with Tourette's.”

### A Place to Share and Ask for Information About Tics

Posts to the online community often consisted of members requesting information and advice. The most common request concerned tic management and triggers of tics. Members frequently asked questions regarding ways to reduce, suppress, or redirect tics (“Does anyone know how to relax facial tics?”); treatment options and their effectiveness and side effects (“I thought about trying to suppress my tics by medication. What are the possibilities? Are there any negative long term affects?”); tic severity and frequency (“Is there any research or information of Tourette’s worsening in adulthood?”); tic triggers (“Can it be that alcohol triggers my tics?”); and suggestibility (“Does reading about other people's tics make yours worse?”). Another common request was for advice regarding the diagnosis of Tourette syndrome. Members often described their symptoms and asked the community whether this was Tourette syndrome or if what they described were actually tics. Community members also asked questions around the benefits or disadvantages of having a Tourette syndrome diagnosis. Information and advice regarding the management of comorbid conditions (most often attention-deficit hyperactivity disorder, obsessive compulsive disorder, depression, and anxiety) were also evident. Some posts came from members who used the community to seek information or advice on ways to support their loved one with the syndrome (“What is the best thing to do for someone who is having an attack?”). Members also requested information and advice regarding a range of Tourette syndrome–related everyday issues, for example, how to talk to loved ones about the condition, employment, discrimination, preventing damage to health from tics, and relationship advice.

In response to requests for information and advice, members often replied with comments giving advice and suggestions. Some community members posted advice or suggestions around what worked for them. Members most frequently shared advice for and experiences with managing tics. Prescription medication (frequently mentioned were muscle relaxants, alpha-agonist hypotensive medications, antipsychotics, and carbonic anhydrase inhibitors) and their benefits and risks were discussed. They also discussed and recommended other treatment options (eg, cannabis, herbal remedies, dietary changes and supplements, stress reduction, massage, music, behavioral therapies for tics) and often would share their methods to replace or suppress tics. Some stated that their tics lessen or completely stop when they do something that they enjoy or something that requires intense concentration (eg, playing a musical instrument). Discussions took place around diagnosis: Members shared their anecdotal experience of getting diagnosed with Tourette syndrome or a tic disorder, advice regarding health care professionals, benefits of a diagnosis (“a diagnosis gives you validation, I didn’t realise how much of a difference it makes!”; “getting diagnosed could save you from a law suit or getting fired”), and reasons for not pursuing a diagnosis (“I feel that my tics aren't a big issue, and I don't want to waste the doctor’s time”). In response to posts in which the member posting requested advice regarding diagnosis or shared unusual symptoms, other members often encouraged the advice seeker to see a health care professional and suggested caution (“We are not neurologists”) when giving advice or information. Community members also shared factual information about Tourette syndrome, tics, and comorbid conditions (eg, diagnostic criteria). They gave advice to loved ones of people with the condition on how to best support them, for example, communicate openly, not reacting to tics, understand and learn more about it, listen, reassure them, and connect them to the Tourette syndrome community. Members also offered advice regarding legal protection of Tourette syndrome as a disability and suggestions on dealing with discrimination (“they have to reasonably accommodate it and can't fire you for it, under the disability law”). They also signposted members to other sources of help or information related to the condition (eg, charities, research articles, health care professionals, other OSCs, TV shows, and films).

### A Place to Unload My Feelings

The online community was frequently used to vent emotions, such as expressing emotional pain and frustration resulting from living with Tourette syndrome, often in long posts or comments, and sometimes indicating that the community member needed to “vent/get it off their chest” (“The struggle is real ... ... It’s a nightmare and I needed to vent”). Community members often expressed empathy and understanding when replying to posts in which others had shared a tic or experience (ie, verbal description or picture/meme), stating that they had similar tics or experiences and can relate and understand their pain (“I feel your pain!”; “I definitely understand that feeling”). Members also offered reassurance (“That's all perfectly normal”; “You will be ok”) and validated other members’ emotions and experiences (“That must be hard to deal with”; “You should be mad. This is not ok.”). Kind wishes (“Good luck!”) were numerous, and members also expressed their sympathies in response to posts or comments disclosing negative experiences (“I’m so sorry! That really sucks!”).

Members encouraged each other to accept their tics (“You also don’t have to hide your tics! ...You’re unique in your own way and you shouldn’t have to hide yourself!”; “Stay twitchy my friend”) as well as general support (“I know you can do it”; “Hang in there”). Members also praised each other for achievements and sharing created artworks (“I am so incredibly proud of you!”). Humor appeared to be one way to cope with Tourette syndrome, as some members reported that they joke with people “in the real world” about it (“I try to make fun of it to see the social anxiety about it and usually it gets a few laughs… dark humour to some but I would rather people laugh at the jokes about my Tourette’s”) and recommended this coping strategy to others. Members also requested and shared uplifting or encouraging “success” stories and expressed delight in the success of others.

### A Place Where I Can Find People Like Me

Community members expressed gratitude to others for the advice and support offered from other users, who understood the kinds of adversity they faced due to having Tourette syndrome (“I just wanted to say I really REALLY enjoy this place. Seeing other people having to grow up and deal with the same I went through is amazing. I knew no one else, so I could never talk about the issues I was dealing with at school etc.”). In these posts and comments, members reached out to members in a variety of ways including offering to connect through private message, sharing personal information, and providing updates to previous posts.

Some negative aspects were also identified. Some members were hostile towards others when they disagreed with posts or comments. Some “gatekeeping” issues were also observed as some members complained about the quantity of posts requesting advice regarding diagnosis (“This community has just become a place to ask if you have Tourette’s rather than discussing tics and how to deal with them”). This possibly led one individual to wonder if they were “allowed” in the OSC, as their Tourette syndrome was undiagnosed. Other users argued that even though the frequent diagnosis-related posts were irritating for some users, “the pros of this remaining a safe place for (mostly) young people to come and ask about TS as it possibly relates to them far outweigh the negatives.”

### A Place Where I Can Freely Share the Realities of Living With Tourette's

Members described or showed their tics or suspected tics. The tics shared included vocal and motor tics, and some members shared what their first tics were and some unusual tics and tic presentations (tics in sleep, paralysis tics, and tic attacks). Some members also shared personal videos of their tics. Members often responded with sharing their own tics, and many commented that seeing or hearing about other members’ tics was comforting and made them “feel better.” Members sought and offered solidarity (“Please let me know I'm not the only weird one in having this tic?”; “we all do our best to help each other out here and no matter what we’re always here if you needs support!”). Premonitory urges and physical issues caused by tics (eg, headache, pain, dental issues, injury) were also discussed.

Members shared their unique realities of living with Tourette syndrome, such as what triggers their tics, how their tics wax and wane, and comorbid conditions with which they live. In some posts, members described how their tics felt like to them, while users expressed their Tourette syndrome experience through art. Members shared their anecdotal experiences of dealing with others' misunderstandings, such as rude remarks, staring, bullying, abuse, accusations of “faking” tics, inadequate Tourette syndrome–related health care, and misrepresentation in the media. The impact of Tourette syndrome on their everyday life was also discussed, for instance, tics limiting people by interfering with schoolwork, everyday activities, social life, or relationships. Members emphasized the importance of in-person social support (“having friends and a partner who doesn't respond negatively to my tics has been very helpful”) and shared some positive support experiences (“at uni I was accepted and understood, accommodated for and never talked down to, or ridiculed”). Members shared their concerns about “passing on” Tourette syndrome and the complexities of family planning (“Me and my wife are trying to have kids ...I don't want my kid to have to deal with tics and all of this... It's a constant moral dilemma”).

Members discussed whether they disclose their tics to other people (“I always just let my co-workers know so that they don't think I'm on drugs or something”; “don't fancy getting them involved so they don't worry. Or maybe …they'd think less of me.”). Members also discussed whether they should embrace or deny their tics (“tics are part of who I am”; “no matter how much I try to accept it, I am unable to”), and some discussed that they are sometimes unsure if they are “faking” having tics or Tourette syndrome (“I’m worried that the doctor will not believe me because even i don't believe myself when i speak about the tics”).

## Discussion

### Principal Findings

To date, few studies have looked at social support within OSCs for individuals living with Tourette syndrome or tic disorders. This study aimed to examine the provision of social support in 1 online Tourette syndrome community through an inductive thematic analysis of postings. The findings of this study suggest that users utilized the online community as a multifaceted virtual place where they could share and ask for information about tics, unload their feelings, find people facing similar situations and experiences, and freely share the realities of living with Tourette syndrome. Compared with studies taking similar inductive approaches to analyzing messages in online support groups [19,38,40,41], this study had a large data set reflecting 10% of all threads made in the community since its inception.

The findings in this analysis appear to align with those of previous studies using similar methodologies and finding that OSCs for several chronic conditions tend to report similar functions of social support, including OSCs as a valued virtual place for informational and emotional support from peers experiencing the same health issue, sharing personal experiences with others with empirical lived understanding, and sharing and “venting” emotional reactions typically common with experiencing a chronic condition [19,38,45]. The results from this study complement the findings from a previous online survey exploring users’ experiences with participating in Tourette syndrome/tic disorder OSCs [47]. What this study adds is that, by analyzing posts created naturalistically over time in 1 group, it has identified additional social support needs unique to tic disorders. For example, the “A place to unload my feelings” theme identified an emotional support need relating to users’ discussions that they may be “faking” their tics or symptoms and dealing with victimization from other people. This may be unique to people with tics, given the nature of tics and the socially stigmatizing nature of tic disorders. These findings may also be of value to health care professionals in further understanding the emotional needs of patients with tic disorders and how they can be supported.

In line with the findings by Meade et al [42], the results of this study suggest that OSCs have a potentially valuable role as a mechanism for sharing and gaining information on illness experiences and empowering individuals and supportive others in relation to self-management of neurodevelopmental conditions such as Tourette syndrome. The online nature of the support community may also aid social support by providing an anonymous environment through which users can disclose information that they would find difficult to express in person [39] or discuss sensitive topics [28]. For people with Tourette syndrome, online communities may provide an accessible and inclusive space, where they can gain social support not easily available in their offline worlds—and from peers who understand what it is like to be socially excluded and “different” due to their tics [47]. Regular online community users may also gain additional benefits as a consequence of providing support to other users, in accordance with the helper-therapy principle [54], which suggests that people also help themselves when helping others, by taking on important social roles, developing their coping skills, and directing their focus away from their own problems.

### Limitations and Future Directions

Despite the insights generated through this study, there are limitations that should be considered. First, our analysis focused on textual data; therefore, conversations between community members did not have any associated nonverbal conversational cues. This arguably makes our task more difficult and creates risks around misinterpretation of the data. These risks are exacerbated by the fact we did not engage community members in the analytical process. However, to mitigate against this risk, each post analyzed was done so in the context of the full conversation thread. Second, this study looked at only 1 Tourette syndrome OSC; therefore, the extent to which the results can be generalized may be limited, as other communities may differ in their structure, membership, community dynamics, and types of social support requested or offered. Third, little is known about the demographic characteristics of the community members included in this study; therefore, it is difficult to assess how representative they are of the wider population of individuals with Tourette syndrome or tic disorders. Additionally, users of the online community were a mix of people with a tic disorder themselves and supportive others (eg, parents, caregivers, partners). It was not always obvious from the posts and threads whether the user was a person with tic disorder themselves or a caregiver or supportive other of someone with tics; therefore, it was not possible to do a subanalysis by type of user. Although not a limitation in itself, there may be some similarities and differences in the benefits and kinds of social support these 2 groups seek online, given their different roles in the illness experience.

Finally, this analysis was conducted on data collected prior to the COVID-19 pandemic. During the pandemic, there have been reports from clinicians regarding increased numbers of referrals to specialist tic clinics or services—particularly from adolescent girls—with some clinicians suggesting this new influx of patients is linked to online video-based media from Tourette syndrome content creators (eg, TikTok, YouTube) [55,56]. Understandably, given this and societal changes during the pandemic (eg, potentially not being able to access usual health care systems or peer support), usage of online communities for Tourette syndrome and tic disorders may have changed or membership increased substantially during this time—and this was not captured in our analysis. Looking at publicly available statistics for the 1 OSC used in this study, as of January 1, 2022, there was a 235% increase in registered users over 2 years. Between its inception and December 31, 2019, there were 5382 threads posted to the OSC; between January 1, 2020, and January 1, 2022, there was a 125% increase in threads posted over 2 years. These data suggest increased membership and usage over the past 2 years, which overlaps with the COVID-19 pandemic.

Given the present study only looked at 1 online support group, future research may wish to explore the social support provided across multiple OSCs for Tourette syndrome and tic disorders. Studies could also explore the online experiences of individuals with Tourette syndrome or tic disorders and caregivers separately, as previous research suggests potential mismatch between these 2 groups of users [47]. Furthermore, this study analyzed the content of online communication, which is arguably in the public domain. It is unknown whether discourse varies in private OSCs, as well as communities developed using a range of different platforms and modalities of communication (eg, Facebook, Discord), and this could potentially be explored in further research.

### Conclusion

Online support may be a useful, easily accessible addition to traditional forms of support for people with Tourette syndrome or tic disorders and their supportive others, where they can share and request information about tics, unload their feelings, find people with similar experiences, and share the realities of living with the condition.

## Abbreviations

NIHR: National Institute for Health and Care Research
OSC: online support community
